# Open Versus Robotic Cystectomy: A Propensity Score Matched Analysis Comparing Survival Outcomes

**DOI:** 10.3390/jcm8081192

**Published:** 2019-08-09

**Authors:** Marco Moschini, Stefania Zamboni, Francesco Soria, Romain Mathieu, Evanguelos Xylinas, Wei Shen Tan, John D Kelly, Giuseppe Simone, Anoop Meraney, Suprita Krishna, Badrinath Konety, Agostino Mattei, Philipp Baumeister, Livio Mordasini, Francesco Montorsi, Alberto Briganti, Andrea Gallina, Armando Stabile, Rafael Sanchez-Salas, Xavier Cathelineau, Michael Rink, Andrea Necchi, Pierre I. Karakiewicz, Morgan Rouprêt, Anthony Koupparis, Wassim Kassouf, Douglas S Scherr, Guillaume Ploussard, Stephen A. Boorjian, Yair Lotan, Prasanna Sooriakumaran, Shahrokh F. Shariat

**Affiliations:** 1 Department of Urology, Comprehensive Cancer Center, Medical University of Vienna, Vienna General Hospital, A-1090 Vienna, Austria; 2 Department of Urology, Urological Research Institute, San Raffaele Scientific Institute, 20132 Milan, Italy; 3 Department of Urology, Luzerner Kantonsspital, Spitalstrasse, 6000 Luzern, Switzerland; 4 Division of Urology, Department of Surgical Sciences, University of Studies of Torino, 10124 Turin, Italy; 5 Department of Urology, Rennes University Hospital, 35000 Rennes, France; 6 Department of Urology Bichat Hospital, Paris Descartes University, 75877 Paris, France; 7 Division of Surgery and Intervention Science, University College London, London WC1E 6BT, UK; 8 Department of Uro-Oncology, University College London Hospital NHS Foundation Trust, London W1T 4EU, UK; 9 Department of Urology, “Regina Elena” National Cancer Institute, 00128 Rome, Italy; 10 Urology Division, Hartford Healthcare Medical Group, Hartford, CT 06106, USA; 11 Department of Urology, University of Minnesota, Minneapolis, MN 55455, USA; 12 Department of Urology, L’Institut Mutualiste Montsouris, Université Paris Descartes, 75014 Paris, France; 13 Department of Urology, University Medical Center Hamburg-Eppendorf, 20251 Hamburg, Germany; 14 Fondazione IRCCS Istituto Nazionale dei Tumori, 20133 Milan, Italy; 15 Cancer Prognostics and Health Outcomes Unit, University of Montreal Health Centre, Montreal, QC H4A 3J1, Canada; 16 Sorbonne Université, GRC n°5, ONCOTYPE-URO, AP-HP, Hôpital Pitié-Salpêtrière, F-75013 Paris, France; 17 Bristol Urological Institute, North Bristol NHS Trust, Southmead Hospital, Bristol BS10 5NB, UK; 18 Department of Urology, McGill University Health Center, Montreal, QC H4A3J1, Canada; 19 Department of Urology, Weill Cornell Medical College, New York-Presbyterian Hospital, New York, NY 10038, USA; 20 Department of Urology, La Croix du sud Hospital, 314000 Toulouse, France; 21 Department of Urology, Mayo Clinic, 200 First Street Southwest, Rochester, MN 55905, USA; 22 Department of Urology, University of Texas Southwestern Medical Center, Dallas, TX 75390, USA; 23 Department of Molecular Medicine and Surgery, Karolinska Institutet, 17177 Stockholm, Sweden; 24 Department of Urology, Weill Cornell Medical College, New York Presbyterian Hospital, New York, NY 10021, USA; 25 Department of Urology, The University of Texas M.D. Anderson Cancer Center, Houston, TX 77030, USA

**Keywords:** bladder cancer, robotic-assisted, open, radical cystectomy, survival, propensity score

## Abstract

Background: To assess the differential effect of robotic assisted radical cystectomy (RARC) versus open radical cystectomy (ORC) on survival outcomes in matched analyses performed on a large multicentric cohort. Methods: The study included 9757 patients with urothelial bladder cancer (BCa) treated in a consecutive manner at each of 25 institutions. All patients underwent radical cystectomy with bilateral pelvic lymphadenectomy. To adjust for potential selection bias, propensity score matching 2:1 was performed with two ORC patients matched to one RARC patient. The propensity-matched cohort included 1374 patients. Multivariable competing risk analyses accounting for death of other causes, tested association of surgical technique with recurrence and cancer specific mortality (CSM), before and after propensity score matching. Results: Overall, 767 (7.8%) patients underwent RARC and 8990 (92.2%) ORC. The median follow-up before and after propensity matching was 81 and 102 months, respectively. In the overall population, the 3-year recurrence rates and CSM were 37% vs. 26% and 34% vs. 24% for ORC vs. RARC (all *p* values > 0.1), respectively. On multivariable Cox regression analyses, RARC and ORC had similar recurrence and CSM rates before and after matching (all *p* values > 0.1). Conclusions: Patients treated with RARC and ORC have similar survival outcomes. This data is helpful in consulting patients until long term survival outcomes of level one evidence is available.

## 1. Introduction

Bladder cancer (BCa) is the second most common genitourinary malignancy with 81,190 estimated new diagnoses for 2018 in the United States alone [1]. Radical cystectomy (RC) with bilateral pelvic lymph node dissection (PLND) is the standard treatment for muscle invasive and very high risk non-muscle invasive BCa [2]. However, this procedure is associated with significant perioperative mortality and morbidity as a direct consequence of the complexity of the procedure and the characteristics of the population which is generally older and suffering from multiple comorbidities when compared to other surgical patients [3]. Minimally invasive surgeries, such as robotic assisted radical cystectomy (RARC), have been designed to improve surgical morbidity. Indeed, robotic-assisted radical surgery in urology has been shown to be associated with decreased blood loss, need for transfusion, and length of stay compared to open RC (ORC) in most studies [4,5,6,7,8,9,10]. 

While these perioperative benefits are generally accepted, the differential impact of RARC compared to ORC on survival outcomes remains debated with widely diverging opinions [4,11,12]. The RAZOR trial [13], a randomized, open-label, non-inferiority, phase 3 trial comparing ORC and RARC, found that RARC was non-inferior to open cystectomy for 2-year progression-free survival but did not report overall survival. 

Given the shortage of prospective randomized trials comparing RARC to ORC, controlled data regarding the oncological risks and benefits are needed from well-designed retrospective multicenter studies. 

Therefore, to address this unmet need, we collected complete data from BCa patients treated at academic centers to determine the impact of on survival outcomes of RARC compared to the standard ORC. We performed a propensity-matched analysis to limit the impact of selection bias on survival outcomes. 

## 2. Experimental Section

### 2.1. Patients and Methods

We collected the data from 9757 patients treated with RC for non-metastatic UCB at 25 institutions. Patients were staged preoperatively with cross sectional imaging (mostly computerized tomography), bone scan when indicated and chest x-ray. Surgical specimens were processed according to standard pathologic procedures at each institution. Tumors were staged according to the 2009 American Joint Committee on Cancer-Union Internationale Centre le Cancer (AJCC/UICC) TNM classification. Tumor grade was assigned according to the 2003 WHO/International Society of Urologic Pathology (ISUP) consensus classification. STSM was defined as the presence of tumor at inked areas of soft tissue on the RC specimen [14,15]. Urethral and ureteral margins were not considered as STSM. Lymphovascular invasion (LVI) was defined as the presence of tumor cells within an endothelium-lined space without underlying muscular walls [16,17].

### 2.2. Primary and Secondary End Points

The primary end-point was to compare survival outcomes of RARC with ORC. The secondary end-point was to evaluate survival outcomes of BCa patients treated with RARC. Overall recurrence and cancer-specific mortality (CSM) were defined as disease recurrence and death from disease, respectively.

### 2.3. Statistical Analyses

Descriptive statistics of categorical variables focused on frequencies and proportions. Means, medians, and interquartile ranges (IQR) were reported for continuously coded variables. The Mann–Whitney and chi-square tests were used to compare the statistical significance of differences in medians and proportions, respectively. Fine and Gray multivariable competing risk analyses tested the impact surgical technique and survival outcomes. Owing to inherent differences between patients undergoing ORC and RARC in terms of baseline patient and disease characteristics, we used a 2:1 propensity score matched analysis to adjust for the effects of these differences. The use of the propensity score method reduces the customary bias associated with the conventional multivariable modeling approach. The variables adjusted for were administration of neoadjuvant chemotherapy (NAC), grade, pathological T stage, lymph node status and age at surgery Subgroup analyses were performed. Statistical significance was considered at *p* < 0.05. Statistical analyses were performed using SPSS v.22.0 (IBM Corp., Armonk, NY, USA) and STATA 14 (Stata Corp., College Station, TX, USA).

## 3. Results

### 3.1. Clinicopathologic Characteristics (Entire Cohort)

Demographics and pathologic characteristics of the cohort stratified by surgical approach are shown in Table 1. Overall, 767 (7.8%) patients were treated with RARC and 8990 (92.2%) with ORC and most of the patients were men (*n* = 7775, 80%); median age was: 68 years (IQR: 60–74). About half of the patients (*n* = 4248, 45%) harbored pathological stage T3-T4, 6.7% had positive STSM (*n* = 639) and 24% (*n* = 2276) had lymph node metastases. There were no differences in age at surgery and gender between RARC and ORC patients (all *p* values > 0.1). Conversely, patients treated with RARC were more likely treated with NAC (26% vs. 3.6%) compared to patients treated with ORC and had less advanced diseases (pT3-pT4 stage: 40% vs. 46% and lymph node metastasis 22% vs. 24%). RARC patients were less likely to receive adjuvant chemotherapy compared to ORC patients (13% vs. 21%). 

### 3.2. Clinicopathologic Characteristics (Adjusted Cohort)

Demographics and pathologic characteristics of the cohort after propensity matching, stratified by surgical approach are reported in Table 2. After the propensity matching, 420 (33%) patients were treated with RARC and 840 (67%) with ORC; no differences were recorded between ORC and RARC patients considering age, gender, NAC usage, pathological T stage, pathologic grade, and lymph node invasion (all *p* > 0.1). On the other hand, patients treated with RARC recorded higher rate of positive STSM compared to ORC group (11% vs. 6.3%).

### 3.3. Survival Analyses in the Entire Cohort (Unadjusted Cohort)

**Figure 1:**
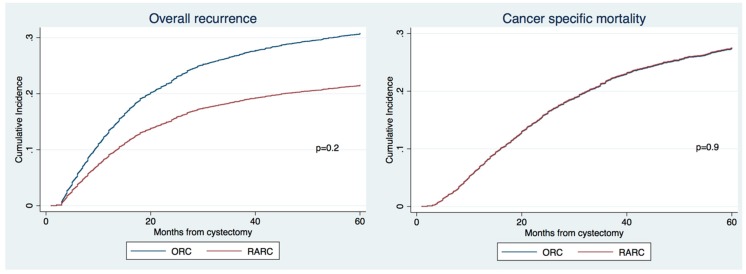
Cumulative incidence of recurrence and cancer specific mortality on overall population of patients with non-metastatic bladder cancer (BCa) treated with radical cystectomy according the type of surgery (ORC vs. RARC).

The median follow-ups before and after propensity matching were 81 and 102 months, respectively. The 3-year recurrence rates, CSM and OM were 37% vs. 26%, 34% vs. 24% and 47% vs. 34% for ORC vs. RARC (Figure 1, all *p* values > 0.1), respectively. On multivariable Cox regression analyses adjusting for standard clinico-pathologic characteristics, no significant differences were found between RARC and ORC in overall recurrence and CSM (Table 3, *p* > 0.1). 

### 3.4. Survival Analyses after Propensity Matching (Adjusted Cohort)

**Figure 2:**
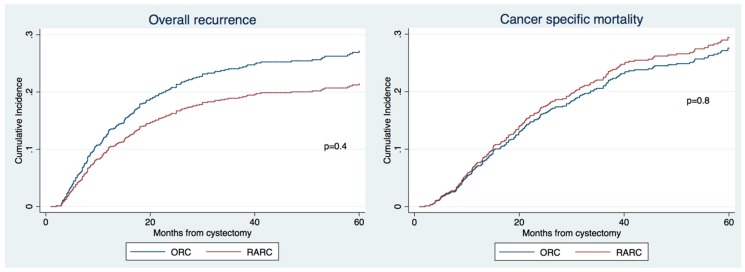
Cumulative incidence of recurrence and cancer specific mortality of patients with non-metastatic BCa treated with radical cystectomy according the type of surgery (ORC vs. RARC) after 2:1 propensity matching for age, pathological T stage, pathological N stage, neoadjuvant chemotherapy (NAC) and grade.

The 3-year recurrence and CSM were 31% vs. 29% and 27% vs. 26% for ORC vs. RARC, respectively (Figure 2, all *p* values > 0.3), respectively. On multivariable Cox regression analyses adjusting for standard clinicopathologic characteristics, RARC was again associated with similar overall recurrence and CSM compared to ORC (Table 4, *p* > 0.3).

## 4. Discussion

The adoption of RARC is growing rapidly, but the majority of radical cystectomies continues to be performed by a conventional open approach. The majority of the current data from RARC series which tested perioperative and short term oncological outcomes did not test equivalence regarding long term survival outcomes [18,19,20,21]. Several retrospective series raised, indeed, some concerns regarding the oncological safety of the robotic approach [22]. On the other hand, two different prospective trials found no differences in survival outcomes between the two surgical approaches [13,23]. 

In this multicenter study, we evaluated the survival outcomes of the largest international cohort of bladder cancer patients treated with either ORC or RARC. Patients were treated in both European and American referral centers, collecting data from almost 1000 RARC and matching them with almost 9000 ORC patients. This manuscript follows two previous publications [10,21] from the same collaboration, evaluating for the first time the impact of survival and on peri-operative outcomes demonstrating an advantage of RARC in blood loss and length of stay. New centers were added to this manuscript in respect of the previous publications and the match of the final database was performed separately for each study on the bases of the main aim of each project. 

We found that RARC and ORC share similar survival outcomes, both on univariable and multivariable analyses controlled for established prognostic factors. We performed propensity matching to minimize the risk of selection bias adjusting for pathological stage, lymph node status, and age at surgery. Even in this setting we confirmed that the RARC approach is associated with similar recurrence and CSM rates compared to ORC. These results were obtained with a median follow-up before and after propensity matching of 81 and 102 months, respectively. Similarly to our previous manuscript [21], we found a positive surgical margin status higher than 10% in patients treated with RARC. However, this was consistently higher than in patients treated with RARC compared to patients treated with ORC. Despite these differences, this had no impact on survival outcomes when adjusted for all the available confounders in the multivariable model. 

Our results confirm the findings of the RAZOR trial [13], an open label, randomized, phase 3, non-inferiority trial comparing RARC versus ORC. A total of 152 patients were included in the ORC group and compared to 150 patients treated with RARC, reporting similar 2-year progression free survival rates. Bochner et al. [23], in a prospective, randomized trial compared 60 and 58 patients treated with RARC and ORC, respectively. No differences were found considering recurrence, cancer survival, or overall survival. Previously, Bochner et al. [18] reported in a single center prospective randomized trial, an advantage in terms of mean intraoperative blood loss for the RARC group but longer operative times compared to ORC. However, no survival outcomes were reported. Similarly, in the prospective trial of Khan et al. [24] and Nix et al. [20] survival outcomes were not analyzed. Given the paucity of prospective data analyzing survival outcomes of RARC patients, new long term level one evidence are required. 

Several retrospective series focused on mid-long-term survival outcomes [22,25]. Nguyen at al. [22] analyzed 383 consecutive patients treated with ORC (120) or RARC (263) between 2001 and 2014 at a single institution. With a median follow up of 30 months (for ORC) and 23 months (for RARC), they reported similar recurrence rates with an increasing risk of experiencing extrapelvic lymph node recurrence and peritoneal carcinomatoses for RARC patients. Our analyses did not include the type of recurrence limiting our ability to test this aspect; but we found a similar overall recurrence risk for patients treated with RARC when compared to ORC. 

Hu et al. [25], using the SEER database compared 439 patients treated with RARC and 7308 treated with ORC. These authors observed an increasing trend in RARC utilization over the study period and with a median follow up of 44 months, they found no survival differences between the two techniques. However, as recognized by the authors themselves, they analyzed only a small RARC cohort treated by many different centers in their learning in some cases. In a recent systematic review and meta-analyses [26], five studies with a total of 540 participants were included. Authors found no differences in disease progression and local recurrences between patients treated with RARC and ORC. Finally, a recent large retrospective study analyzed the outcomes of RARC versus ORC in the selected population of patients who had received perioperative chemotherapy (in the neoadjuvant or adjuvant setting). No difference was found in multivariable analyses in the rate of positive surgical margins, rate of neobladder diversion, recurrence, and overall survival [27]. 

Our study represents the largest multicenter collaboration analyzing survival outcomes of patients affected by bladder cancer analyzing the effect of the RARC approach. Our analyses differentiate itself from previous reports including referral centers but excluding low case volume and learning curves which may lead to suboptimal outcomes. Our study comprises the largest available cohort to date analyzing survival outcomes in RARC patients. Despite several strengths, our study is not devoid of limitations. First and foremost, we recognize that our study is limited by its observational nature, and thus our results should be interpreted within the limits of its retrospective design. Second, we did not perform a central review of all specimens and therefore relied on the dedication and attention of the local uro-pathologists. Third, we did not include data regarding urinary diversion that might have an influence on survival outcomes. On the other hand, previous literature failed to prove any differences regarding different urinary diversion in RARC patients supporting the hypothesis of similar survival outcomes between these two groups [28]. Patients treated in academic centers are more prone to be treated with RARC as compared to ORC [8], moreover, differences exist regarding tumor characteristics, patient characteristics, and year of surgery (with an increasing tendency to perform a RARC) [29,30]. These elements can only partially be adjusted for with a propensity match analysis; we are aware that our results need to be confirmed in a controlled randomized trial. In this regard, a high proportion of RARC patients were found with pT0 disease at RC specimen, that might indicate a selection bias that can be only partially mitigated by the propensity matching analyses. 

## 5. Conclusions

Patients treated with RARC were found with an increased rate of positive surgical margin compared to those treated with ORC. However, no differences regarding overall recurrence rate and survival were found between the two study groups. These results were confirmed in propensity score matched analyses adjusted for all the major confounders. High quality prospective trials are warranted to support the long-term oncological safety of RARC. 

## Figures and Tables

**Table 1:** Clinicopathologic demographics of 9757 patients with bladder cancer treated with radical cystectomy according type of surgery.

Variables	Overall (*n* = 9757, 100%)	RARC (*n* = 767, 7.8%)	ORC (*n* = 8990, 92%)	*p* Value
Age, years				
Mean	67	67	67	0.2
Median (IQR)	68 (60–74)	68 (62–74)	68 (60–74)	
Gender				
Male	7775 (79%)	612 (80%)	7163 (80%)	0.9
Female	1981 (20%)	115 (20%)	1827 (20%)	
Neoadjuvant chemotherapy	520 (5.3%)	198 (26%)	322 (3.6%)	<0.001
Pathological T stage				
pT0-pT1	2908 (31%)	368 (48%)	2540 (29%)	<0.001
pT2	2239 (24%)	93 (12%)	2146 (25%)	
pT3-pT4	4248 (45%)	305 (40%)	3943 (46%)	
High grade	8734 (94%)	361 (76%)	8373 (94%)	<0.001
LNI	2276 (24%)	158 (22%)	2118 (24%)	0.001
Nodes removed, number				
Mean	20	21	20	0.001
Median (IQR)	16 (10–26)	20 (13–28)	16 (9–25)	
Positive surgical margins	639 (6.7%)	107 (10.0%)	532 (6.3%)	<0.001
LVI	3007 (33%)	25 (27%)	2982 (34%)	0.2
Adjuvant chemotherapy	1828 (19%)	85 (13%)	1743 (20.9%)	<0.001

RARC: robotic assisted radical cystectomy, ORC: open radical cystectomy, IQR: interquartile range, LNI: lymph node invasion, LVI: lymphovascular invasion.

**Table 2:** Clinicopathologic characteristics of 1374 patients with bladder cancer treated with radical cystectomy, comparing robot assisted radical cystectomy (RARC) and open radical cystectomy (ORC) cohorts after propensity matching.

Variables	Overall (*n* = 1374, 100%)	RARC (*n* = 420, 33%)	ORC (*n* = 840, 67%)	*p* Value
Age, years				
Mean	66	66	66	0.9
Median (IQR)	67 (59–73)	67 (61–72)	67 (51–72)	
Gender				
Male	1003 (80%)	365 (80%)	728 (79%)	0.9
Female	257 (20%)	93 (20%)	188 (21%)	
Neoadjuvant chemotherapy	456 (33%)	1162 (35%)	294 (32%)	0.2
Pathological T stage				
pT0-pT1	535 (39%)	189 (41%)	346 (38%)	0.4
pT2	208 (15%)	52 (11%)	156 (17%)	
pT3-pT4	631 (46%)	217 (47%)	414 (52%)	
High grade	1075 (78%)	348 (76%)	727 (79%)	0.1
Nodes removed, number				
Mean	19	22	17	0.001
Median (IQR)	16 (10–25)	19 (14–28)	14 (8–24)	
LNI	318 (23%)	109 (24%)	209 (23%)	0.6
Positive surgical margins	115 (8.4%)	52 (11%)	63 (7.0%)	0.006
LVI	302 (32%)	23 (45%)	282 (32%)	0.04
Adjuvant chemotherapy	211 (16%)	45 (12%)	166 (18%)	0.004

RARC: robotic assisted radical cystectomy, ORC: open radical cystectomy, IQR: interquartile range, LNI: lymph node invasion, LVI: lymphovascular invasion.

**Table 3:** Multivariable competing risk analyses predicting the risk of overall recurrence and cancer specific mortality (CSM) in patients treated with radical cystectomy in overall patients.

Variables	Overall Recurrence		CSM	
	HR (CI 95%)	*p* Value	HR (CI 95%)	*p* Value
Gender (male vs. female)	1.07 (0.97–1.17)	0.1	1.15 (1.04–1.27)	0.005
Age, years	1.00 (0.99–1.00)	0.5	1.00 (0.99–1.00)	0.052
RARC approach	0.65 (0.34–1.26)	0.2	1.00 (0.45–2.24)	0.9
pT stage				
pT0-pT1	Ref	Ref	Ref	Ref
pT2	1.35 (1.18–1.55)	<0.001	1.49 (1.27–1.73)	<0.001
pT3-4	2.10 (1.84–2.40)	<0.001	2.62 (2.27–3.03)	<0.001
pN+	1.68 (1.51–1.86)	<0.001	2.09 (1.88–2.33)	<0.001
Nodes removed	0.99 (0.99–1.00)	0.3	0.99 (0.99–1.00)	0.04
High grade vs. low	2.53 (1.73–3.71)	<0.001	2.37 (1.56–3.60)	<0.001
LVI	1.44 (1.31–1.57)	<0.001	1.33 (1.21–1.46)	<0.001
Positive surgical margins	1.43 (1.25–1.65)	<0.001	1.64 (1.42–1.90)	<0.001
Neoadjuvant chemotherapy	1.69 (1.36–2.10)	<0.001	1.45 (1.15–1.85)	0.002
Adjuvant chemotherapy	1.18 (1.06–1.31)	0.001	0.89 (0.80–0.99)	0.03

CSM: cancer specific mortality, HR: Hazard ratio, CI: confidence interval, RARC: robotic assisted radical cystectomy, LVI: lymphovascular invasion.

**Table 4:** Multivariable competing risk analyses predicting the risk of overall recurrence and CSM in patients treated with radical cystectomy after propensity matching.

Variables	Overall Recurrence		CSM	
	HR (CI 95%)	*p* Value	HR (CI 95%)	*p* Value
Gender (male vs. female)	1.09 (0.80–1.48)	0.6	1.23 (0.91–1.67)	0.1
Age, years	1.00 (0.99–1.01)	0.5	1.01 (0.99–1.02)	0.09
RARC approach	0.76 (0.39–1.47)	0.4	1.34 (0.49–2.36)	0.8
pT stage				
pT0-1	Ref	Ref	Ref	Ref
pT2	1.21 (0.77–1.90)	0.3	1.34 (0.84–2.15)	0.2
pT3-4	1.57 (1.04–2.37)	0.03	2.17 (1.40–3.35)	<0.001
pN+	1.43 (1.05–1.94)	0.02	2.33 (1.71–3.16)	<0.001
Nodes removed	0.99 (0.98–1.00)	0.3	0.98 (0.97–0.99)	0.01
High grade vs. low	3.20 (1.55–6.59)	0.002	3.60 (1.62–7.98)	0.002
LVI	1.85 (1.37–2.49)	<0.001	1.27 (0.96–1.70)	0.09
Positive surgical margins	1.12 (0.74–1.69)	0.5	1.30 (0.84–2.01)	0.2
Neoadjuvant chemotherapy	1.96 (1.51–2.54)	<0.001	1.34 (1.02–1.76)	0.03
Adjuvant chemotherapy	1.29 (0.94–1.77)	0.1	0.77 (0.56–1.06)	0.1

CSM: cancer specific mortality, HR: Hazard ratio, CI: confidence interval, RARC: robotic assisted radical cystectomy, LVI: lymphovascular invasion.
